# Supplementary material for: Transplantation of human iPSC-derived microglia ameliorates neuropathology and circuit dysfunction in progranulin-deficient mice
Source: Mol Neurodegener. 2026 Jul 16;21:64. doi: 10.1186/s13024-026-00978-6 (PMC13656582; doi:10.1186/s13024-026-00978-6)
Supplement: Supplementary file 1 [file 13024_2026_978_MOESM1_ESM.docx]

# **LIST OF SUPPLEMENTARY MATERIALS**

**Figures S1–S6** description and figures included below

**Table S1:** List of marker genes, DEGs, and upstream pathway analyses for xenotransplanted human microglia. Related to **Figure 3.**

**Table S2:** List of marker genes and DEGs for hippocampal mouse astrocytes and oligodendrocytes. Related to **Figure 4.**

**Table S3:** List of marker genes and DEGs for thalamic mouse astrocytes and oligodendrocytes. Related to **Figure 4.**

**Table S4**: List of marker genes and DEGs for thalamic mouse excitatory neurons and inhibitory neurons. Related to **Figure 5.**

**Table S5:** Human control and FTD patient information and lists of marker genes and DEGs for human patient excitatory neurons and inhibitory neurons. Related to **Figure 5.**

# **Figure S1. Progranulin protein levels within peripheral organs and the brain**. Levels of murine progranulin within the liver (t=27.60, ^****^*p* < 0.0001) **(A)**, plasma (t=96.25, ^***^*p* =0.0004) **(B)**, and spleen (t=7.07, **p* = 0.0192) **(C)** of *hCSF1-WT* and *hCSF1-Grn^–/–^* littermates were measured by ELISA. N = 3 mice/group, unpaired two-tailed t-test. **(D)** Progranulin levels measured within the brain (t=10.31, ^**^*p =* 0.0015). N = 4–5 mice/group, respectively, unpaired two-tailed t-test. **(E)** Progranulin levels measured within the brain using a human-targeted ELISA, n = 5 samples/group, one-way ANOVA with Tukey’s multiple comparisons test (F_2,12_=1.803, ^****^*p <* 0.0001, ^*^*p=*0.0287). **(F)** Progranulin levels measured within the brain using a mouse-targeted ELISA, n = 5 samples/group, one-way ANOVA with Tukey’s multiple comparisons test (F2,12=2.80, ^****^*p <*0.0001).

# **Figure S2. Mouse cytokine levels in the brain.** A multiplex ELISA was used to concurrently examine protein levels of several murine cytokines and chemokines within the brains of *hCSF1*-PBS, *hCSF1-Grn^–/–^* +PBS, and hCSF1-Grn***^–/–^*** +xMG engrafted mice. For each analyte, N = 10 mice/group were compared by one-way ANOVA with Tukey’s multiple comparisons test. No significant (ns) differences in IL-6 **(A)** or KC/GRO **(B)** were detected. **(C)** IL-9 levels (F_2,27_= 4.411, ^*^*p* = 0.02). **(D)** IL-15 levels (F_2,27_ = 18.69, ^***^*p* = 0.0002 and ^****^*p* < 0.0001). **(E)** IL-17A/F levels (F_2,27_ = 20.64, ^****^*p* < 0.0001). **(F)** IP-27p/28 levels (F_2,27_ = 16.73, ^***^*p* = 0.0006 and ^****^*p* < 0.0001). **(G)** MCP-1 levels, ns = nonsignificant. **(H)** MIP-2 levels (F_2,27_ = 6.54, ^*^*p* = 0.0156 and ^**^*p* = 0.0083). **(I)** IL-2 levels, ns = nonsignificant. **(J)** IL-12p70 levels, ns = nonsignificant. **(K)** IL-5 levels (F_2,27_ = 4.009, ^*^*p* = 0.0367).

# **Figure S3. Xenografted human KOxMG or WTxMG exert differential effects on mouse hippocampal astrocyte and oligodendrocyte transcriptomes**.

# **(A)** Volcano plot of pseudobulk differentially expressed genes (DEGs) in hippocampal astrocytes between WTxMG and KOxMG groups shows significant upregulation and downregulation of multiple genes in the KOxMG group.

# **(B)** Top predicted upstream regulators identified using Ingenuity Pathway Analysis (IPA).

# **(C)** Heatmap of complement related genes.

# **(D)** Representative confocal microscopy of xenografted GFAP-positive (white), SOX9 (red) in all experimental groups; scale bar: 100 µm.

# **(E)** Quantification of SOX9 (nuclei/FOV) in the hippocampus of wild-type and transplanted mice showing no significant differences between any groups; *p* values from one-way ANOVA (Hippocampus: F_2,41_ = 0.07708; *p*=0.9259) with Tukey’s multiple comparisons tests. Data represented as mean value ± SEM (non-injected *hCSF1* littermates, n=15; *hCSF1-Grn^-/-^* + PBS, n=14; *hCSF1-Grn^-/-^* + xMG, n=16) (Female, n=8 per group; male, n=6-8 per group).

# **(F)** Quantification of GFAP (volume) in the hippocampus of wild-type and transplanted mice; *p* values from one-way ANOVA (Hippocampus: F_2,41_ = 3.269; ^*^*p*=0.0482) with Tukey’s multiple comparisons tests. Data represented as mean value ± SEM (non-injected *hCSF1* littermates, n=15; *hCSF1-Grn^-/-^* + PBS, n=14; *hCSF1-Grn^-/-^* + xMG, n=16) (Female, n=8 per group; male, n=6-8 per group).

# **(G)** Quantification of GFAP/SOX9 cell ratio in the hippocampus of wild-type and transplanted mice; *p* values from one-way ANOVA (Hippocampus: F_2,41_ = 0.8583; *p*=0.4314) with Tukey’s multiple comparisons tests. Data represented as mean value ± SEM (non-injected *hCSF1* littermates, n=15; *hCSF1-Grn^-/-^* + PBS, n=14; *hCSF1-Grn^-/-^* + xMG, n=16) (Female, n=8 per group; male, n=6-8 per group).

# **(H)** Volcano plot of pseudobulk DEGs in hippocampal oligodendrocytes between WTxMG and KOxMG groups highlights significant transcriptional differences in the KOxMG group.

# **(I)** Quantification of average cell ratios in each subcluster by group in hippocampal oligodendrocytes.

# **(J)** Correlation between Subcluster 2 and Disease Associated Oligodendrocyte genes, R=0.5, p<2.2 e-16.

**Figure S4. QC of sn-RNAseq analysis of hippocampus engrafted with** ***GRN^–/–^* or *GRN^+/+^***

**human microglia.**

**(A), (B)** Quality-control plots showing equivalent amounts of total RNA features and total number of RNA counts, respectively.

**(C)** Violin plot showing percentage of mitochondrial genes detected per nucleus for each individual sample. **(D)** UMAP showing labeling of all cell types in the hippocampus.

**(E)** Dot plot showing expression of identity markers for each cell type.
**(F)** Proportion of each cell type within each genotype.

**(G)** Proportion of each cell type within individual samples. **(H)** Correlation between UMI counts and percentage of mitochondrial genes detected (left) or total gene counts (right) per nucleus for each individual sample.

**Figure S5. QC of sn-RNAseq analysis of thalamus engrafted with *GRN^–/–^* or *GRN^+/+^***

**human microglia.**

**(A), (B)** Quality-control plots showing equivalent amounts of total RNA features and total number of RNA counts, respectively.

**(C)** Violin plot showing percentage of mitochondrial genes detected per nucleus for each individual sample. (**D)** UMAP showing labeling of all cell types in the thalamus.

**(E)** Dot plot showing expression of identity markers for each cell type.

**(F)** Proportion of each cell type within each genotype.

**(G)** Proportion of each cell type within individual samples.

**(H)** Correlation between UMI counts and percentage of mitochondrial genes detected (left) or total gene counts (right) per nuclei for each individual sample.

**Figure S6. Grooming and social interaction behaviors in *hCSF1-Grn^–/–^+*KOxMG and *hCSF1-Grn^–/–^* +WTxMG mice.**

**(A)** Grooming episodes as seen from a side view. Mice groomed both their head and body with front and hind limbs.

**(B)** Grooming episodes snapshots as a single video frame corresponding to schematics in B. Open circles denote grooming limbs, crossed—non-grooming.

**(C)** Quantification of total grooming time, *p* value from Student’s t-test (t(17) = 1.868, *p* = 0.079).

**(D)** Quantification of the number of grooming episodes, *p* value from Student’s t- test (t(17) = 0.1506, *p* = 0.8820).

**(E)** Quantification of the distance traveled, *p* value from Student’s t- test (t(17) = 1.042 *p* = 0.3118).

**(F)** Quantification of the velocity, *p* value from Student’s t- test (t(17) = 1.019, *p* = 0.3227). Data represented as mean ± SEM (*hCSF1-Grn^–/–^+*KOxMG, n = 8; *hCSF1-Grn^–/–^* +WTxMG, n= 11).

**(G)** Percent time spent in the center of the open field arena, Student's t-test (t(17) = 0.06290, p = 0.9506).

**(H)** Interaction episodes of social approach as seen from a side view. Mice approach the pencil wire cup with a 1) snout touch, 2) snout touch and one front limb touch, and 3) rearing (both front limbs touch).

**(I)** Interaction episodes snapshots as a single video frame corresponding to schematics in (G). Crossed circles denote snout, open—front limbs.

**(J–N)** Independent age-matched cohort comparison of grooming behavior in wild-type (WT) and *hCSF1* mice.

**(J) Number of grooming episodes, Student's t-test (t(13) = 0.02818, p = 0.9779).**

**(K)** Total grooming time, Student's t-test (t(13) = 0.2029, p = 0.8423).

**(L)** Mean grooming episode duration per mouse, Student's t-test (t(13) = 0.1114, p = 0.9130).

**(M) I**ndividual grooming episode durations pooled across mice (n = 60 episodes from WT, 53 from hCSF1), Student's t-test (t(111) = 0.3419, p = 0.7330).

**(N)** Cumulative probability distribution of grooming episode durations, fit with an exponential plateau model and compared by extra sum-of-squares F test (F(3, 27) = 2.432, p = 0.0868).

n = 8 WT mice and 7 hCSF1 mice.
